# The Magnitude and Impact of Food Allergens and the Potential of AI-Based Non-Destructive Testing Methods in Their Detection and Quantification

**DOI:** 10.3390/foods13070994

**Published:** 2024-03-25

**Authors:** Akinbode A. Adedeji, Paul V. Priyesh, Adeniyi A. Odugbemi

**Affiliations:** 1 Department of Biosystems and Agricultural Engineering, University of Kentucky, Lexington, KY 40546, USA; 2 Department of Animal and Food Science, University of Kentucky, Lexington, KY 40546, USA; paul.v@uky.edu; 3 Archer-Daniels-Midland Company, Decatur, IL 62526, USA; adeniyi.odugbemi@adm.com

**Keywords:** food allergens, ELISA, biosensors, non-destructive method, celiac, eliciting doses, gluten detection

## Abstract

Reaction to food allergens is on the increase and so is the attending cost on consumers, the food industry, and society at large. According to FDA, the “big-eight” allergens found in foods include wheat (gluten), peanuts, egg, shellfish, milk, tree nuts, fish, and soybeans. Sesame was added to the list in 2023, making the target allergen list nine instead of eight. These allergenic foods are major ingredients in many food products that can cause severe reactions in those allergic to them if found at a dose that can elicit a reaction. Defining the level of contamination that can elicit sensitivity is a work in progress. The first step in preventing an allergic reaction is reliable detection, then an effective quantification method. These are critical steps in keeping contaminated foods out of the supply chain of foods with allergen-free labels. The conventional methods of chemical assay, DNA-PCR, and enzyme protocols like enzyme-linked immunosorbent assay are effective in allergen detection but slow in providing a response. Most of these methods are incapable of quantifying the level of allergen contamination. There are emerging non-destructive methods that combine the power of sensors and machine learning to provide reliable detection and quantification. This review paper highlights some of the critical information on the types of prevalent food allergens, the mechanism of an allergic reaction in humans, the measure of allergenic sensitivity and eliciting doses, and the conventional and emerging AI-based methods of detection and quantification—the merits and downsides of each type.

## 1. Introduction

Food allergy is reported to affect 3–11% of adults depending on the region, and 8% of children worldwide [1,2]. The incident of allergen contamination in foods could have a catastrophic effect on consumers, such as anaphylaxis reactions and death in severe cases, and its occurrence can negatively impact return on investment food manufacturers. The annual economic cost of food allergies in the US in terms of medical and caregiver opportunity cost is estimated to be between $19 and $25 billion [3,4,5]. A proper mitigation effort can significantly reduce the human and economic losses associated with allergic reactions.

In Japan, a group of six foods (chicken egg, cow milk, wheat, shellfish, fruit, and buckwheat) are reported to be responsible for over 75% of the food allergy cases [6]. However, in Europe and the United States, eight classes of food account for over 90% of the most severe food allergic reactions in [7,8]. The eight include wheat (proteins including gluten), peanuts, egg, shellfish, milk, tree nuts, fish, and soybeans [9]. The United States of America’s congress passed the Food Allergy Safety, Treatment, Education, and Research (FASTER) Act in 2021, adding sesame to the list of allergens. This became effective on January of 2023, making the list of food allergens the “big-nine” that must be included in food labels when they are included in foods. The list of allergens controlled by the regulatory agencies differs by country or region. For example, the European Union list extends beyond the big-nine, as it also includes 14 food items, namely cereals containing gluten (such as barley, oats, and wheat), celery, crustaceans (e.g., crabs, prawns, and lobsters), mustard, fish, lupin, eggs, molluscs (e.g., oysters and mussels), milk, peanuts, soybeans, sesame, sulphur dioxide, and sulphites [10]. There are other (170) less serious allergens in foods that cause reactions [11]. It is important to note that not all are common in human diet and not all of them persist to adulthood or cause severe reactions. However, any degree of allergenic reaction could create significant discomfort that could be life-threatening and lead to economic loss at several scales. The most effective way to curtail allergic reactions is through preventive measures such as detection to ensure other foods are not cross-contaminated with them [12].

Allergic reactions to food occur when someone consumes foods that elicit a reaction. This could occur because of a lack of diagnosis or unconscious consumption, often through the cross-contamination of foods with allergens in pre- and post-harvest processing: in-field during harvesting or in-plant where the same processing facility is used for making multiple products or during home processing. 

While the prevention of cross-contamination is critical, what is more important is the proper detection of possible contamination on food processing surfaces and in products in shared facilities or at home front. Regulatory agencies such as the US Food and Drug Administration (FDA) have been enacting laws for over a century to reduce incidents of allergen contamination but are still finding the need to keep updating the list and making new laws, especially pertaining to accurate labeling and detection standards. In a recent news release by the FDA [9], they reiterated their commitment to continue to set standards that will help protect consumers against the improper labeling of foods that may contain allergens. Approximately one-third of foods reported to the FDA through the Reportable Food Registry as serious health risks involved undeclared food allergens. They recognized the risk posed by the inefficient monitoring and labeling of allergenic foods, and they continue to crack down on defaulters. Since March 2020 and January 2021, the FDA sent warning letters to eight big, registered food plants in the US that distributed undeclared major food allergens that resulted in Class I recalls and forced them to implement measures to prevent re-occurrence. Finding problems before they can potentially harm consumers is crucial to the work of the regulatory agencies. The bedrock of effective monitoring and enforcement by regulatory agencies like the FDA and USDA is access to analytical tools that provide a rapid result without the drudgery of chemical assay in most conventional methods for assessing and detecting allergens. Organizations such as the FDA are always looking for new and innovative approaches to make their job easier. 

As part of the attempt by the food industry to increase the throughput and safety of foods, the implementation of Industry 5.0-digital solutions such as non-destructive technologies is said to be essential. Food companies with potential allergen issues are also seeking effective, rapid, and non-destructive methods to detect and determine the allergen levels in their products before they get into the supply chain. This is predicated on the fact that the cost of recall, penalty, and the lawsuit that could follow is significant; it could be as much as 15% of a company’s annual revenue. Companies and regulatory agencies need simple tools (e.g., handheld devices) to assess food and systems for quick feedback. Consumers need to be able to assess foods themselves on the spot [13]. A celiac patient should be able to determine if a product contains sufficient gluten to harm them before they make a purchasing decision. These are all critical needs that highlight the importance of developing non-destructive methods for allergen detection and quantification. The current tools in allergen detection are efficient but are mostly chemical or biochemical assays that require sample preparation, are slow in providing feedback, and lead to the production of wastes. However, the current advances in machine and deep learning have led to the development of a higher accuracy of pattern recognition and quantitative-non-destructive determination technologies for a minuscule level of biological and chemical material constituents in foods through interactions with light and sensors. The major limitation is the high cost of implementation, which is currently being addressed through innovative solutions that are making the method become increasingly affordable. Sensor and machine learning coupling in allergen detection is a classic example of artificial intelligence (AI) deployment within the food supply chain with the capability to increase the sustainability of the allergen detection method and reduce the human involvement and response time for reliable feedback. 

This review paper looks at some of the current topics on food allergens, considers the challenges posed by food allergen sensitivity, highlights the importance of detection and quantification, and introduces the promise of emerging non-destructive methods leveraging sensors and machine learning in contrast to conventional approaches.

### 1.1. Food Allergenicity 

Food allergy is a public health problem that is reported to be on the rise especially in industrialized countries and with a high prevalence in children [1,14]. Food allergies and other types of food hypersensitivities affect millions all over the world and their families. It occurs when the body’s immune system reacts to certain proteins in food, and in rare cases, carbohydrates such as galactose-α-1,3-galactose (α-gal) [15]. The mechanism of an allergic reaction is such that the body’s immune system has mistaken certain food constituents as harmful. This immune response occurs in two stages. The first phase is sensitization to an allergen and the second is the elicitation of an allergic reaction on subsequent exposure to the same allergen. Sensitization occurs when a susceptible individual produces immunoglobulin E (IgE) antibodies against specific proteins in a food. When re-exposed to the same food, the allergenic proteins bind to IgE molecules on immune mediator cells (basophiles and mast cells), leading to the activation of these mediator cells. This elicitation causes the release of inflammatory molecules (e.g., leukotrienes and histamine) [16]. The reaction could vary from mild symptoms, like swollen lips, edema, and hives, to severe reactions that could be life-threatening symptoms like anaphylaxis. Food allergies are increasing because of the changes in our diet and better diagnostic methods [17,18]. CDC in the US reported a 50% increase in the prevalence of food allergy in children between 1997 and 2011 [14,19]. 

The best cure for allergic reaction is prevention [3]. The FDA wrote, “Finding problems before they can potentially harm consumers is a critical element of the FDA’s allergen enforcement program” [9]. This starts from being able to identify the foods or allergens that an individual reacts to and ensure that they avoid such in their diet. There are nine common food allergens (big-nine) that are notorious (causes 90% of all food allergies) for causing allergic reactions in humans. They include wheat (gluten), peanuts, egg, shellfish, milk, tree nuts, fish, sesame, and soybeans [9]. This is where proper detection is critical. It is imperative that there are arrays of techniques to quickly detect food allergens before they cause harm to consumers. The FDA requires that all food ingredients are properly labeled and those that contain allergens are labeled as such. Unfortunately, many of the foods in our stores are not meeting this labeling requirement. For example, in one European study, peanuts were found in 25% of cookies and 43% of chocolate labeled with the precautionary phrase “may contain”. More concerning was that 25% of chocolate and 11% of cookies without advisory labeling tested positive for peanuts [20]. In another example, the FDA found evidence of milk in 75% of chocolate products with advisory labeling and evidence of milk even in products without advisory labeling or specifically with dairy-free claims [21,22]. Since 2020, the “Whole Foods Market” has recalled more than 30 food products due to the presence of major food allergens which were not properly labeled and were discovered through an FDA inspection [9]. 

### 1.2. Reference Doses 

Determining the threshold doses of allergens below which subjects are safe is critical in developing evidence-based technologies that ensure the safety of consumers. This is the typical amount consumed at one sitting that elicits a reaction immediately or up to 30 min afterwards [23]. For example, a food considered to be gluten-free must contain less than 20 ppm [<20 mg/kg] of gluten [24]. In 2011, the Allergen Bureau of Australia and New Zealand (ABA) worked to establish the References Doses (RDs) for 11 allergenic foods to guide food labeling. The data were pulled from across the world, including the US and Europe. The RDs (mg of the total protein from the allergenic foods that elicit an allergic reaction) were determined in 1% (cow milk and peanut) and 5% (wheat, soybean, cashew, shrimp, sesame seed, mustard, and lupin) of the allergic population depending on the food. The specific RDs for peanut and milk were found to be 0.2 and 0.1 mg, respectively, at 1% eliciting doses (ED_01_). The RDs for wheat, soybean, and shrimp were 1.0, 1.0, and 10 mg at ED_05_, respectively [25]. In another effort by a European Commission group (EuroPrevall project) to build on the Australian–New Zealand standardization of allergens’ threshold, they conducted a double-blind, placebo-controlled food challenge (DBPCFC) study to define the threshold for five allergens for 10% of the allergic population (ED_10_), and they found the threshold as 2.8 mg of peanut protein (or the consumption of 11.2 mg of peanut that contains about 25% protein), 9 mg of hazelnut protein (67 mg of hazelnut; 13.7% protein [dry weight]), 1.6 mg of celeriac powder (100 mg of raw celeriac root; 1.5% protein wet weight), 27 mg of fish (152 mg of raw codfish flesh; 17.8% [wet weight]), and 2.5 g of shrimp (12.8 g of raw shrimp flesh; 20.3% [wet weight]) [26]. Recently, two groups from the Netherlands (TNO) and Food Allergy Research and Resources Program (FARRP) at the University of Nebraska-Lincoln, USA, applied Bayesian Stack modeling, a novel statistical approach that derives a single outcome based on different models while accounting for the degree of fit of the various models. With this new approach, they harmonized previous data points and updated the threshold dose distribution analyses for 14 allergenic foods and published doses (at 95% confidence intervals) that are predicted to elicit mild objective allergic symptoms in 1% (the Eliciting Dose 01 or ED_01_) and 5% (ED_05_) of the allergic population [12]. The new ED_01_ and ED_05_ values for wheat, milk, peanut, egg, shrimp, and soy among the 14 allergens are 0.7, 0.2, 0.2, 0.2, 26.2, 0.5 mg and 6.5, 2.4, 2.1, 2.3, 280, and 10.0, respectively [12,23]. The lack of an international consensus (except gluten regulated by Codex Alimentarius) on the threshold permissible to allergic reactions has made it hard to develop effective and universal labeling and detection technologies [27]. 

The conventional methods of detecting and quantifying food allergens are mostly chemical assay methods. A lot of these chemical methods are sensitive, accurate, and robust. However, their drawbacks include the drudgery of sample preparation, waste production, need for trained personnel, and the fact that they are often not rapid enough and are destructive methods. However, there are emerging non-destructive methods that are capable of being sensitive, robust, accurate, rapid, and easy to use by non-technical personnel.

### 1.3. Food Allergy by Age 

Milk is the most common food allergy for children below the age of two with a prevalence of 2–6%, while egg accounts for some prevalence seen in children [8]. However, allergic reactions change over a lifetime. Some develop allergies as adults [8]. While allergic reactions to milk, egg, wheat, and soy typically resolve during childhood, allergies to peanuts, gluten, tree nuts, fish, and shellfish persist to adulthood [14]. The treatment regime and therapy vary but most people would live a life avoiding foods that they are allergic to, and this is why effective detection methods are critical to help most consumers to avoid foods that could cause them harm. Below, we profiled two common allergic reactions—wheat/gluten and peanut allergies. 

### 1.4. Examples of Common Food Allergens

#### 1.4.1. Wheat and Gluten Allergens 

Wheat allergy is defined as an adverse immunological reaction that is caused by the proteins found in wheat [28]. Gluten is the main structural protein of wheat, composed of two main fractions depending on their solubility in aqueous alcohols: the monomeric soluble gliadins and the poorly soluble glutenin [29]. These reactions are not only due to gluten, but may also be triggered by other proteins found in wheat including albumins (water-soluble and hardened by heat) and globulins (dissolves best in salt solution) [30]. The symptoms associated with the reactions may rapidly progress from tolerable to acute symptoms. Approximately 7% of the American population react to the protein in wheat, including gluten. There are three types of wheat-related reactions, namely celiac (gluten intolerant), wheat allergy, and non-celiac gluten sensitivity (NCGS). The first two are classified as allergic reactions to proteins in wheat, while the third, NCGS, is considered a non-autoimmune non-allergic reaction [31]. One in every 133 Americans (2.47 million) is reported to be suffering from celiac while the global prevalence is put at 5% [31]. Gluten that causes celiac is also present in other grains like barley and rye as homologous proteins [32]. 

In celiac disease (CD), a T-cell mediated autoimmune reaction is triggered by gluten-derived peptides. This autoimmune inflammatory cascade is localized in the small bowel, where it leads to the classical enteropathy and malabsorption syndrome that creates severe stomach pain in patients [32]. The cost of treating celiac in the first and second year in the US is about $10,815 and $4712, respectively [33]. Just like other allergens, wheat allergenic reaction does not have a cure; the best cure is prevention, and it is imperative that consumers are able to make an informed choice when selecting their diet to ensure they are wheat/gluten-free. The conventional method for wheat and gluten detection includes using the polymerase chain reaction (PCR) and ELISA methods [34,35], which are accurate and reliable but come with the drudgery of analysis, need for trained personnel, and waste that pollutes the environment.

The question of how processing such as thermal treatment affects the efficacy of food antigens also arises. The baking of wheat flour-containing foods results in the loss of IgE binding to one group of recognized wheat allergens, the alpha-amylase inhibitors. However, baking does not affect the ability of wheat prolamin to bind IgE from wheat-allergic individuals [36]. The implication of this is that thermal processing can reduce wheat protein-related allergenicity to some degree but not completely [37,38]. Hence, there is a need for a non-destructive means to ascertain the level of wheat protein in thermally processed foods.

#### 1.4.2. Peanut Allergenicity 

Peanuts, just like wheat, are a common food allergen. Its prevalence is on the rise because of the widespread use of peanut products, which has made food processors to be more proactive in their responsibility in preventing peanut contamination by implementing good manufacturing practices (GMPs) and allergen control programs based on proven technologies/regimes that effectively monitor allergen cross-contamination. Reports indicate that peanut allergies cause most of the annual emergency department admissions from food allergies and up to 63% to 67% of deaths caused by anaphylaxis [39,40]. Unlike most other types of food allergies such as milk, egg, wheat, and soy that resolve in childhood, peanut allergies (PAs) persist in 75–80% of children into adulthood [41]. PAs affect between 1% and 2% of the global population and about 7–14% of PA patients will experience accidental peanut exposure annually [42]. This makes its attending economic cost very high. A 2017 white paper found that patients with Pas averaged approximately 1.25 medical services per patient in 2016 based on an analysis of nationwide US medical insurance claims, with an average charge per visit at $236.73. This amount excludes the cost of lost labor, medication, special diets/allergen-free foods, and changes in schools, and annual opportunity costs due to forgone labor market activities. The fatality and cost related to PAs underpin the importance of prevention which borders on an adequate and reliable detection method. This is the more reason for simple to use, easily accessible, and effective PA monitoring systems that an average consumer can use to reduce incidents of contact with their attending economic cost.

### 1.5. Conventional Methods for Allergen Assessment

Currently, most of the standard techniques for the detection of food allergens are analytical procedures that demand high costs, require skilled technicians, include long sample preparation times, and involve the production of by products that constitute an environmental waste disposal problem [43,44]. These standard methods include the use of lab-scale analytical devices, such as high-performance liquid chromatography (HPLC) or gas chromatography–mass spectrometry [45]. Another approved effective analytical method for detecting and quantifying allergens in food is the enzyme-linked immunosorbent assay (ELISA) [24]. 

#### 1.5.1. Enzyme-Linked Immunosorbent Assay (ELISA) 

ELISA is a unique analytical method for the detection of food allergens through the recognition and binding of species-specific antigens by specific antibodies [46]. This method, apart from being costly, time-consuming, and difficult to perform, requires a high level of wet-chemistry skills [47]. An alternative to the direct detection of proteins using ELISA is the polymerase chain reaction (PCR), which is a DNA-based method to target molecules with specific DNA sequences [48]. While the PCR is a highly specific and sensitive alternative method to detect allergens in food, it is also not suited for rapid on-site detection because it is too time-consuming, laborious, and costly [49]. It is also not easily accessible to all consumers who may want to test their foods for potential allergens. 

#### 1.5.2. Mass Spectrometry (MS) 

MS is another method that has been increasingly exploited to determine allergens in food because of the technique’s capacity for multiplexing and providing unequivocal allergen identification [50]. The method relies on monitoring proteolytic peptides selected as markers of food allergens that are mostly protein. The MS method, like other chemical assays, is reliable, specific, and accurate, but comes with significant preparation and a need for an expensive equipment that needs high technical skill to operate coupled with a slow feedback time. 

#### 1.5.3. Biosensors 

**Figure 1:**
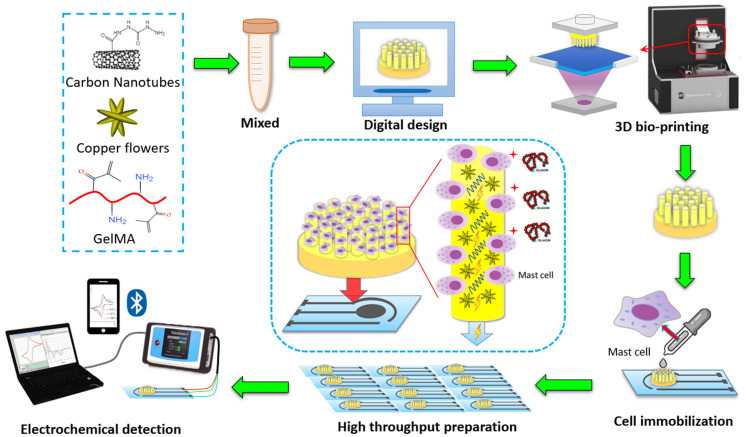
Schematic drawing of cell-based electrochemical biosensor for gliadin (wheat protein) detection–assembly and procedure [55]. Copyright from Elsevier.

Biosensors are a group of measurement techniques that can provide a relatively accurate, fast, qualitative, and quantitative detection of allergens in an easy and portable way [44]. Biosensors are analytical instruments containing a biological sensing element or a biomimetic material, such as antibodies, nucleic acids, aptamers, enzymes, proteins, microorganisms, cells, or tissues, for the quantitative or qualitative detection of target analytes [51,52]. Typically, biosensors include four main components: (a) a recognition element or bioreceptor, (b) transducer, (c) detector, and (d) display unit. Generally, biosensors are categorized based on their transducer type as electrochemical, optical, or mechanical biosensors [53]. However, among these biosensor types, only the optical ones have the capacity of being used for on-site measurements in a remote manner [44]. Electrochemical biosensors, which are the most used subcategories due to their higher sensitivities and possibility to be miniaturized and integrated into high-density arrays, require the tested samples to be disposed [45]. Figure 1 shows the complexity of the steps needed in using an electrochemical biosensor for the detection of gliadin (gluten protein). Mechanical biosensors, on the other hand, are less common in food safety and quality assessment because of the high complexity and costs associated with their fabrication. Overall, biosensors are currently rarely used in the food industry and need more research to improve their sensitivity (lower limit of detection, LOD), specificity, stability, and fabrication costs. The development of nano-biosensors provide great potentials to overcome these challenges in the future [54].

## 2. Non-Destructive Detection of Allergens in Foods

Non-destructive food testing comes with the added advantage of being robust, rapid, and easy to use. They combine these with high accuracy, reliability, and accessibility. Examples include the hyperspectral imaging (HSI) technique, computer vision, electronic nose, and Fourier transform infrared (FTIR) spectroscopy. Their uniqueness lies in their ability to non-destructively evaluate test samples without leading to waste, and the materials can be further used as food if not contaminated. Some of them can be deployed into phone APPs for easy accessibility by consumers. Some of the relevant examples are discussed below. 

### 2.1. Fourier Transform Infrared (FTIR) Spectroscopy 

**Figure 2:**
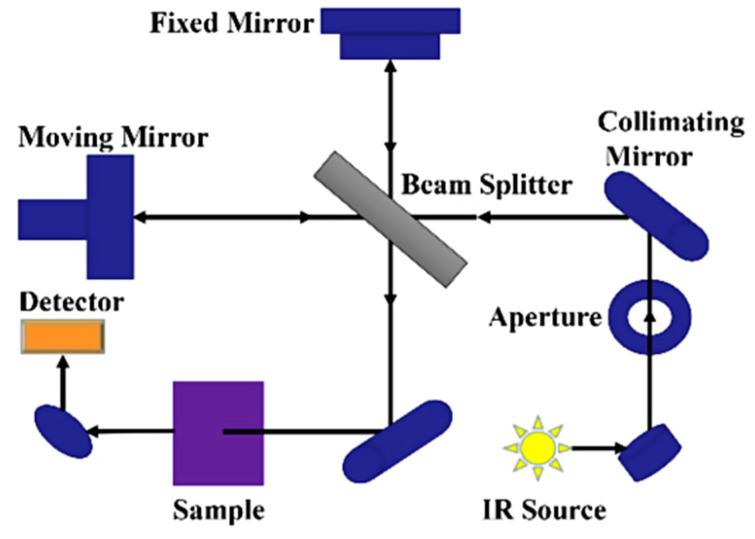
Schematic diagram of FTIR spectrometer [57].

FTIR spectroscopy is a rapid partially non-destructive technique based on the infrared light interaction with the molecular vibration of bonds in organic, inorganic, and polymeric materials to acquire the chemical and structural information of the sample being tested [38,56]. Light is made to pass through a sample to be tested and the functional groups within the sample absorb specific infrared frequencies (Figure 2). This absorption shows up as peaks in the FTIR spectrum. The baseline principle for FTIR spectroscopy is that molecules absorb specific frequencies of infrared radiation. The infrared radiation absorbed by a molecule corresponds to the vibrational energy levels of its bonds, representing the energy associated with the absorbed infrared radiation. Within a spectrum obtained for a sample, specific regions (wavenumber, cm^−1^) are synonymous with certain chemical bonds, and this is what is depicted in the FTIR spectrum. For example, the entire FTIR spectrum is divided into two distinct groups, the functional and fingerprint groups.

The functional group ranges between 4000 and 1500 cm^−1^, and the fingerprint group is <1500 cm^−1^ [58]. The region between 3500 and 3200 cm^−1^ corresponds to the OH bonds, and the region between 1700 and 1400 cm^−1^ is synonymous with the amide (NH) bonds (protein) [59,60,61]. The exact positioning of the absorption bands and their intensity within each subregion is a function of the specific functional group and the surrounding chemical environment. This is where the application of machine learning models for pattern recognition is critical in accurately identifying compounds by region and intensity when scanned with FTIR. In a study conducted by Adedeji, Okeke, and Rady [61], they developed a rapid method (multispectral models and APP for FTIR data) for the detection and quantification of the cross-contamination of corn flour and its baked product with gluten using the FTIR system coupled with advanced machine learning methods. They intended to demonstrate the effectiveness of FTIR as a rapid and non-destructive method for gluten allergen detection in foods.

**Figure 3:**
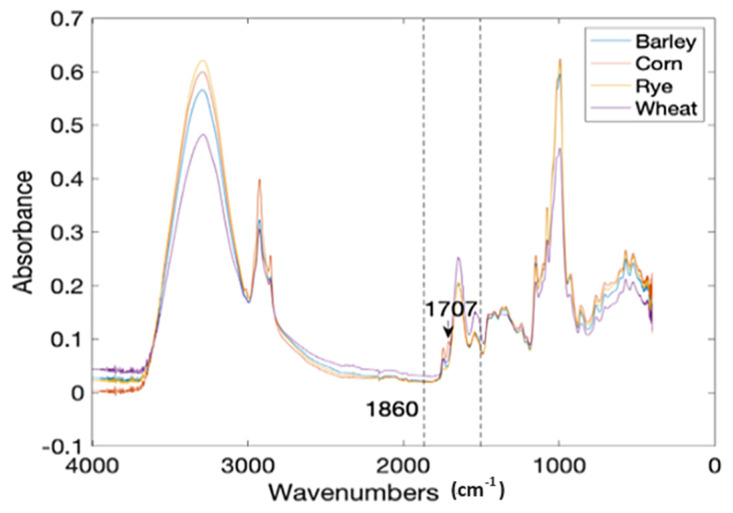
The mean FTIR spectra of four common flour samples [37].

Corn flour (CF) was deliberately contaminated with gluten from three sources that include wheat (WF), barley (BF), and rye (RF) at a 0.5% to 10% level with a 0.5% increment. Each treatment level was prepared in 10 replicates, including pure CF, WF, BF, and RF. This gave a total of 640 treatments scanned for output (Figure 3). The attenuated total reflectance (ATR) spectra of the samples were recorded on a FTIR spectrometer (Nicolet™ iS50, Thermo Fisher, Waltham, MA, USA) in the frequency range of 4000–450 cm^−1^ with a resolution of 4 cm^−1^ and a total accumulation of 32 scans. The data per treatment were then divided into calibration and prediction sets at a ratio of 4:1 (80% to 20%) using the Kennard-Stone (KS) algorithm [62]. That is, 80% of the data was used for training and 20% for the testing or validation of the models. The spectral region between 1860 and 1480 cm^−1^ (C-N, C-C, C=O stretching vibrations) was selected as the region of interest for the classification models because they are synonymous with the protein infrared spectrum and amide I and amide II bands [63] (Figure 3). The data were preprocessed by the Savitzky–Golay (SG) method (best performing preprocessing technique) to eliminate noise.

**Figure 4:**
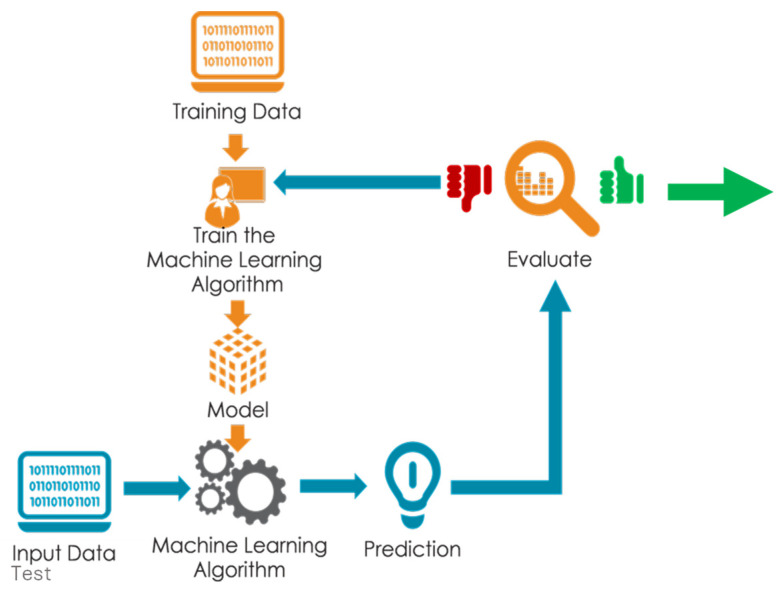
Sketch of spectral data analysis step using machine learning model prototyping.

Different machine learning classifiers such as k-nearest neighbors, decision trees, and linear discriminant analysis (LDA) were applied to the preprocessed data for the initial training model development (Figure 4). The SG-LDA model coupled with bootstrap bagging was selected as the best classifier based on the confusion matrix results (Table 1). The first 80% of the data was divided into four equal parts for a four-fold cross-validation and the remaining 20% of the data was used to test the model on new data not used for building the model. An attempt was also made to predict the wheat gluten contamination of baked cornbread using a majority voting-based ensemble learning and ML approach. The result for the test set data for the CF contaminated with WF, BF, and RF indicates very few misclassifications of just 2 out of 40 levels of contamination between 0.5 and 10% (Table 1) and very high F1 scores between 0.949 and 1, which is a measure of classification accuracy. The partial least square regression (PLSR) result of prediction for CF contamination quantity with WF, BF, and RF gave a coefficient of determination (R^2^) and root mean square error of prediction for the cross-validation (cv) 0.98, 0.94, 0.98, and 0.82, 0.99, 0.53, respectively. The preprocessing methods most suitable for each gluten source are Mean Centering, Smoothing 2nd Derivative, and Robust Auto-Scaling, respectively.

(1) $$\begin{array}{l} Y_{BF}=9.09-3979.99X_{2193.18\;{cm}^{-1}}-146.97X_{2192.70\;{cm}^{-1}}\\ \;\;\;\;\;\;+4810.34X_{2192.22\;{cm}^{-1}}+2196.16X_{2159.91\;{cm}^{-1}}\\ \;\;\;\;\;\;-2976.17X_{2159.46\;{cm}^{-1}}-182.63X_{2155.58{cm}^{-1}}\\ \;\;\;\;\;\;-4321.57X_{2155.10\;{cm}^{-1}}+7891.61X_{2025.40{cm}^{-1}}\\ \;\;\;\;\;\;-3058.68X_{2024.92\;{cm}^{-1}} \end{array}$$

Equation (1) presents the regression model based on the wavenumber features selected within the FTIR spectrum for corn flour contaminated with barley flour (BF). It is possible to build an APP (such as the Glutini APP from the lead author’s group based on the TensorFlowLite platform) that has the capability of being coupled with ML models (Equation (1)), where data from the FTIR scan can be loaded and analyzed to return the contamination percentage and ppm concentration. This is a very useful tool for ordinary consumers to use in checking their foods for potential gluten allergen.

FTIR has several applications that include the identification of unknown materials through the various functional group peaks that predominate its spectrum. It can be used to analyze the purity of a material. Just as was applied by Adedeji et al. [61], it can be used for the quantification of components in a food mixture. It is a useful tool in chemical reaction monitoring and in determining the structure of polymers. A major limitation to the application of FTIR is that it requires some level of sample preparation with some samples, and it is a destructive method in other instances. It is more effective for crystalline or well-ordered samples. It is a challenge to provide a reliable result when the analyte or target compounds are in low concentrations. Some suggested solutions to these limitations include combining FTIR with other techniques such as NMR [64,65], and using the partial least square regression analysis method that could account for complex relationships between spectral features and concentration, leading to more accurate quantification [66]. 

### 2.2. Hyperspectral Imaging

**Figure 5:**
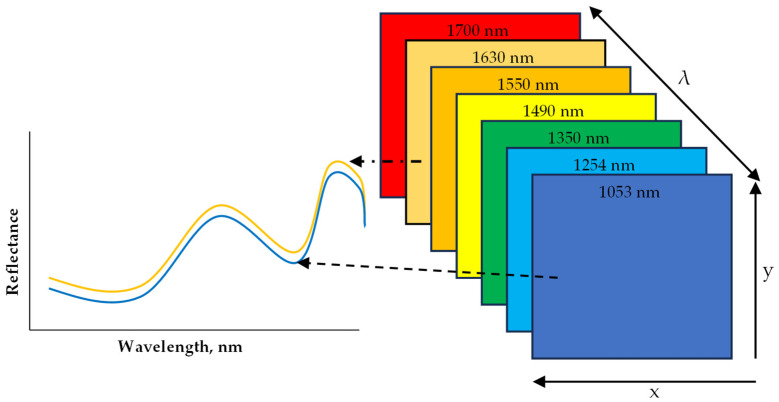
A diagram representation of hyperspectral imaging hypercube within the near infrared (NIR) range.

Hyperspectral Imaging (HSI) has been applied as a sensing technique to non-destructively evaluate the quality of several foods including grains, flours, fruits, meat, vegetables, and processed foods. What is unique about HSI is that it combines conventional imaging and spectroscopy to obtain both spatial and spectral data from an object in a non-destructive way [67,68,69]. The obtained data are arranged into what is called a hypercube. A hypercube comprises an array of x and y spatial data that make up a matrix of vertical and horizontal pixels, while a third axis, λ, represents a spectral dimension (wavelength) (Figure 5). Depending on the resolution of the image, each pixel in the x and y dimension has a reflectance or absorbance at each wavelength within the spectrum, which is spaced at an interval that results in no less than 100 bands and is referred to as HSI [70]. With the added spatial dimension, spectral information can be obtained per pixel of the scanned sample. This in particular makes HSI suitable for the analysis of samples with heterogeneous constituents [69]. The main advantage of an HSI system is the capability to combine spectral features resulting from the interaction between light photons and chemical compounds (from spectroscopy) with spatial features (from traditional vision systems). This also includes the merit of being able to scan and produce a spectrum of individual pixels, which makes it possible to map the desired chemical content or physical defects on a visual image after data processing and modeling. The data analytics steps are similar to that of the FTIR described above. The data acquisition mode often applied is the line (pushbroom) scanning that is more amenable to inline scanning. The preprocessing algorithms and the machine learning models are applied to either classify or predict based on regression with conventional method data. HSI can remotely capture both spatial and spectral information from the regions of interest (ROI) to create a chemical map of the sample for the localization of specific components in heterogeneous food samples. Machine learning (ML) empowered HSI models, while calibrated and upgraded to multispectral models, can detect and quantify the target chemical components in a non-destructive, rapid, and selective way. The model developed can also be deployed as firmware in electronic systems (phone APPs, inline systems) coupled with sensors and feedback systems for automation systems to detect allergens in foods or on food processing surfaces.

A major drawback of HSI is the risk it portends when there is a higher level of false negatives even when the accuracy is very high. Wrongly predicting that a food is contaminant (for example, allergen)-free could have serious implications for the consumers and the food manufacturer. The process of improving the strength of predictions is an on-going step. There is also the high initial cost of implementation. Newer HSI technologies are more cost effective.

### 2.3. Computer Vision (CV) 

CV is a type of non-destructive method that applies the unique physical characteristics of objects captured in a visual image to build predictive models for quality evaluations. CV is a form of artificial vision tools and methods that allow for the acquisition, processing, and analyzing of real-world images in a way that they can be processed by computer systems. Providing machines with the necessary information to make accurate decisions enables them to perform a wide variety of tasks autonomously that include sorting, object differentiation, packaging, and an automated system for food processing activities beyond sorting [71,72]. 

**Figure 6:**
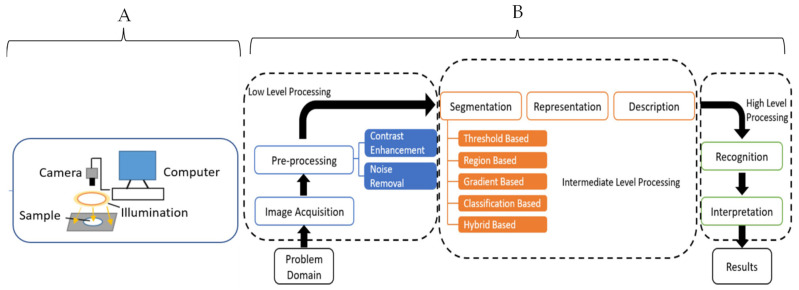
Schematic diagrams for a typical computer vision system (**A**) and data analysis steps (**B**) [71]. Copyright by Elsevier.

CV makes use of powerful optical sensors and machine learning to build algorithms capable of prediction. These include features such as color, shape, texture, and size to evaluate for physical defects such as bruises, insect infestation, senescence damage, ripening stage, classes of objects by size and shape, contaminants detection, variety classification, morphological abnormalities sensing, internal defects, and texture prediction [73]. The steps for data preprocessing and machine learning applications are similar to the steps described for the FTIR (Figure 6). CV differs from HSI in that it focuses on the spatial data, but the baseline principles for data analysis are the same with the HSI technique.

CV is limited in its ability to evaluate the chemometrics of food materials. Nevertheless, it remains a powerful technique for the non-destructive testing of food quality and has some useful applications in allergy detection. The artificial neural network (ANN) is a major machine learning technique for wrangling complex spatial data with a high level of accuracy, among other ML techniques [71]. Rady et al. (2021) demonstrated the use of CV for adulterants’ (that include soy-based products) detection in processed beef meat with a high regression factor (R^2^) of 98% [74]. 

## 3. Prospect for the Future 

The discussed non-destructive methods for the food allergen detection above are scarcely being applied at the industrial level. Despite the vast variety of sensors and methods developed for food evaluation during the last decades. The transfer of the current technologies from laboratory research to commercial food systems is still impeded by some limitations. These include the cost of the technology, concerns about perfect accuracy, perception of complexity, limited industry awareness, and resistance to change. A position paper like this provides a highlight of what is available, their capabilities, limitations, and accessibility. The concerns about accuracy may be a trade-off with regard to the drudgery and slow response in the conventional methods, which could be a last resort if further confirmation is needed. In addition, the current advances in optical sensing and ML allow for the development of powerful non-destructive systems with a higher level of accuracy compared to the destructive test with a limited false positive or negative. Most non-destructive methods are based on electromagnetic energy interactions with foods. Advanced machine learning methods like deep learning and ensemble methods have allowed higher accuracy and more reliable models for pattern recognition that reveal constituents even to the microscale [75]. 

## 4. Conclusions

The increase in allergy diagnosis among consumers and the attending cost of food contamination with allergens necessitate more effective tools in their detection and quantification. Conventional techniques such as the ELISA, mass spectrometry, and biosensors will continue to be important in allergens’ detection and quantification because of their high accuracy, especially in the laboratory testing context. However, the emergence of non-destructive methods, such as computer vision, FTIR, and hyperspectral imaging, will increase the accessibility of testing tools for ordinary customers and stakeholders across the food supply chain. The added advantage of being a sustainable technology with minimal waste production, quick feedback, and reliability that continues to be improved could increase non-destructive technology applications across the food systems, especially in allergen detection.

## Figures and Tables

**Table 1:** Test data model confusion matrix for LDA + 4-fold CV + Bagging [38].

Predicted Classes	Actual Class			
	Class 1	Class 2	Class 3	Class 4
Classified as Class 1	39	2	0	0
Classified as Class 2	0	37	1	0
Classified as Class 3	1	1	39	0
Classified as Class 4	0	0	0	40
Classified as Unassigned	0	0	0	0

Class 1: CF contaminated with BF, Class 2: CF contaminated with WF, Class 3: CF contaminated with RF and Class 4: Pure CF (BF: Barley Flour, WF: Wheat Flour, RF: Rye Flour, CF: Corn Flour), LDA: Linear discriminant analysis. CV–cross validation.

## Data Availability

The original contributions presented in the study are included in the article, further inquiries can be directed to the corresponding author.
